# The acceptance of Covid-19 tracking technologies: The role of perceived threat, lack of control, and ideological beliefs

**DOI:** 10.1371/journal.pone.0238973

**Published:** 2020-09-11

**Authors:** Anna Wnuk, Tomasz Oleksy, Dominika Maison

**Affiliations:** 1 The Robert B. Zajonc Institute for Social Studies, University of Warsaw, Warsaw, Poland; 2 Faculty of Psychology, University of Warsaw, Warsaw, Poland; Middlesex University, UNITED KINGDOM

## Abstract

New technological solutions play an important role in preventing the spread of Covid-19. Many countries have implemented tracking applications or other surveillance systems, which may raise concerns about privacy and civil rights violations but may be also perceived by citizens as a way to reduce threat and uncertainty. Our research examined whether feelings evoked by the pandemic (perceived threat and lack of control) as well as more stable ideological views predict the acceptance of such technologies. In two studies conducted in Poland, we found that perceived personal threat and lack of personal control were significantly positively related to the acceptance of surveillance technologies, but their predictive value was smaller than that of individual differences in authoritarianism and endorsement of liberty. Moreover, we found that the relationship between the acceptance of surveillance technologies and both perceived threat and lack of control was particularly strong among people high in authoritarianism. Our research shows that the negative feelings evoked by the unprecedented global crisis may inspire positive attitudes towards helpful but controversial surveillance technologies but that they do so to a lesser extent than ideological beliefs.

## 1. Introduction

The Covid-19 pandemic (declared by WHO on 11 March 2020) poses an enormous threat to global health, the economy and human well-being. The disease, which first broke out in Wuhan, China in mid-December 2019, has spanned the globe and now exceeds 15,500,000 cases in more than 200 countries, with a death toll reaching 630,000 (as of 24.07.2020). Currently, the USA has become the epicentre of the Covid-19 pandemic, with almost one-third of all cases now being identified in that country. In line with World Health Organization statements that mitigating the impact of Covid-19 should be a top priority for governments, most countries have taken large-scale action to slow down the spread of the disease. Most governments are restricting movement in various ways, from flight cancellations and reinstated border controls to recommending self-isolation for healthy people and enforcing quarantine for all those found to be infected with the virus. In some cases (as in Spain, Italy and France), absolute quarantine was introduced during which leaving home was allowed only for buying medicine or essential products. Large gatherings were forbidden, public events cancelled and school and universities closed and encouraged to provide classes online. To justify imposing such restrictions and limitations on activities and freedom, many countries have introduced the state of emergency. Such drastic measures are also having a significant impact on the job market, with many businesses closing and workers losing wages while governments try to counteract the potential economic crisis via financial support for companies and other special regulations, e.g., delaying taxes and suspending loan payments (see e.g., [1, 2]). The psychological consequences of the epidemic are not yet known, but many mental health experts warn of the potential negative effects of social isolation, permanent uncertainty and dramatic changes to personal plans, not to mention realistic fears of being infected (see e.g., [3]).

Taking the above side effects of lockdowns into account, some countries are seeking other measures to counteract the Covid-19 pandemic. Among these, new technologies play an important role. In France, for example, the police have begun monitoring parks and public spaces with drones to know people do not leave their homes for non-essential purposes. Some governments (e.g., in Israel and Singapore) have gone further and are using smartphone applications that enable tracking those with whom the users have contact to detect the spread of the virus while others (e.g., in Taiwan) have introduced an electronic system that alerts the local authorities if a quarantine obligation is violated (see e.g., [4, 5]). Also, artificial intelligence is often used to predict the spread of the virus and to examine a vast amount of personal data related to Covid-19. For example, Chinese tracking systems used personalised location data combined with facial recognition technology to identify suspected coronavirus carriers or citizens who were not wearing a face mask in public spaces (see e.g., [5]). First reports say that such tracking systems may be effective in counteracting the Covid-19 pandemic when introduced to a large extent (see e.g., [6, 7]).

Although these technologies seem to be effective, their wide implementation raises concerns about privacy and civil liberties violations [8, 9]. Many privacy and human rights advocates warn that the developed surveillance systems could go far beyond monitoring the spread of disease and that the data collected now could be used later for commercial purposes (see e.g., [10, 11]). In non-crisis times, these measures would probably be considered even more problematic in modern democracies, but now they are often perceived as not only useful but also necessary to containing the spread of Covid-19. The present situation can be understood in terms of the so-called ‘state of exception’ ([12], which is triggered by a necessity that assumes that the violation of certain laws is justified to preserve the existing order. In such a state, it is possible to temporarily introduce an exception to existing rights (e.g., freedom of assembly, freedom of movement or some aspects of privacy) to preserve the security of citizens. A quite recent example of a situation in which the rights of individuals were contrasted with the common security was the widely discussed consequences of the terrorist attacks of September 11, 2001 in the USA, when the US government was simultaneously praised for its reaction to the threat and criticised for the ‘generalization of the state of exception’ via excessive monitoring, control and surveillance [13]. It seems that, during the pandemic, the discussion of the state of exception and its potential consequences has become global.

What, then, drives people to accept potentially dangerous surveillance technologies in the effort to protect against the coronavirus? Do perception of the perceived threat and one’s own coping skills during the pandemic influence support for controversial technological solutions? Are these variables related to the COvid-19 pandemic more important than general attitudes towards authoritarianism or liberty?

The main aim of our research was to examine whether the acceptance of technologies that pose a potential threat to privacy and civil rights depends on the situational sense of personal control in a time of pandemic and perceived personal threat as well as on individual differences in authoritarianism, endorsement of liberty and political views.

## 2. Sense of personal control

One of the central human motivations is the pursuit and maintenance of personal control [14]. Individuals experiencing a sense of personal control feel that they are effectively able to influence important aspects of their environment and to steer events in their present and future [14, 15]. Seeing the connection between one’s efforts and what happens in one’s life can provide meaning in an often chaotic world and prevent the unpleasant feeling of randomness [16, 17] As a result, the feeling of control is often associated with numerous benefits, including physical and mental well-being, productivity and happiness (e.g., [18, 19]. On the other hand, a subjective lack of control increases anxiety and depression (e.g., [20, 21]).

The Covid-19 pandemic can certainly be regarded as an extreme threat to the sense of personal control, one even more serious than previous global events linked to a decline in control, such as financial crises (e.g., [22, 23]). First, Covid-19 poses a threat to one’s very existence (or that of relatives), a kind of threat that, according to much research, is deeply interrelated with a lack of control [17, 24]. The epidemic seems to be difficult to counteract, either by individual actions (for example, people cannot be sure that those they meet are not infected), governments (as the public is witnessing ongoing disputes over the most effective ways to counter the epidemic and its consequences) or science (the epidemiology of the virus is still not fully understood, and cautious forecasts indicate that at least a year’s work is needed to develop a vaccine) [25]. Together with financial concerns, incomplete access to information and the indefinite necessity of isolation and social distancing, the above factors may lead to a generalised sense of uncontrollability. Numerous studies have shown that control deprivation inspires attempts to restore it, either by increased personal effort (i.e., primary control) [26] or by seeking sources of external control (e.g., by believing in external powers) [17, 26, 27]. In a time of crisis of the magnitude of the Covid-19 pandemic, people may be even more desperate to reduce the unpleasant feeling of uncontrol via indirect measures, for example, by relying on social norms [28] or endorsing in-group membership [29, 30]. The potential external source of control is often the government, an institution that represents one’s own group and has the capacity to restore the order and predictability of the world. Kay et al. [17] show that people exhibit an increased tendency to rely on social systems when their levels of personal control are low. This *compensatory control model* assumes that formal social systems, such as governments, can provide ‘rules, guidelines, norms and structures’ as religious systems do, which can help people in their quest for control. According to the authors, however, this way of regaining control could be related to increased acceptance of governmental control, which could be particularly visible during crises [14, 28].

The above-mentioned studies demonstrate that, when personal control is threatened, people tend to restore their sense of certainty by adhering to those in power and those with agency (e.g., government and an interventionist God). It is plausible that the acceptance of new types of surveillance technology constitutes an example of an indirect tool for regaining a sense of control. Many such technologies enable the establishment of strict rules that are hard to evade, and, therefore, they may fulfil the need for guidelines and structures. Their advanced technology may also be perceived as ensuring success in the fight against the pandemic. However, so far no research has examined whether lack of personal control would be related to acceptance of measures of control that also pose a threat to privacy.

## 3. The perceived threat of Covid-19

Perceptions of a lack of personal control can be strongly interconnected with the justified concerns experienced by individual during a crisis, e.g., worrying about one’s own life and livelihood or the safety of loved ones [30, 31]. Such concerns are common experiences during the Covid-19 pandemic [32, 33] and can be classified as *realistic threats*, defined as general concerns for one’s well-being [34]. While realistic threats are often related to a personal lack of control, they reflect diverse needs. The first need relates to the immediate risk of losing important resources (including one’s life) and the second to the threat of being unable to cope with the situation (see, e.g., [31]). Research has shown that perceived threats predict attitudes (e.g., [32, 35, 36]), so we examined whether and how the threat of Covid-19 inspires greater support for potential surveillance technologies.

Previous studies on the consequences of perceived threats on attitudes towards civil rights, conducted mainly in the context of terrorist attacks, have shown that people under threat are more likely to accept restrictions on democratic procedures and to exchange their rights for security (see, e.g., [37, 38]). For example, the perception of a threat that a person or a loved one could die in a terrorist attack was shown to increase support not only for stricter methods of dealing with terrorists [38, 39] but also for the harsher punishment of social norm breakers [40], increased surveillance [41] and limiting the freedom of speech [42].

On the basis of previous findings, we hypothesised that the level of personal threat related to the Covid-19 pandemic would inspire greater support for the implementation of anti-epidemic technologies that involve a trade-off between civil rights and security.

## 4. Right-wing authoritarianism

People’s reactions to surveillance have also been associated with their ideological views, specifically with adherence to authoritarianism. Right-wing authoritarianism (RWA) is considered to be an ideological response intended to reduce high levels of perceived threat and anxiety [43, 44], and it has been shown that a perceived terrorist threat predicts stronger support for surveillance among authoritarian people [45]. RWA is also one of the key psychological variables that explain support for increased governmental control and surveillance. It is defined as a submission to authority, adherence to traditional views and hostile behaviour towards groups that violate legitimised rules. Previous research has shown that RWA is positively related to acceptance of human rights restrictions and counterterrorism surveillance measures [45–50]; for a review see [51].

Authoritarian people may thus be more inclined to support surveillance technologies because they are usually introduced as government-led solutions and, as such, promote compliance with norms and obedience to authority. Previous outcomes suggest that RWA beliefs should strengthen the relationship between perceived personal threat and lack of control and a person’s attitude towards surveillance technology. Thus, we tested whether this relationship is particularly strong amongst people high in authoritarianism.

## 5. Endorsement of liberty

When considering the individual factors that may predict attitudes towards such technological solutions, individual endorsement of liberty and freedom must also be taken into account (see e.g., [45, 52]). Research suggests that, under threat, people are generally more willing to accept restrictions on their freedom (e.g., personal emails being scanned and phone calls being recorded) to enjoy a sense of security [50, 52, 53] and that, if they value security, they are not concerned about privacy (see also [54]).

On the other hand, people who are characterised by a strong endorsement of individual liberty often reject other moral considerations, meaning they will resolve the conflict between security and civil rights unequivocally in favour of the latter (see, e.g., [55]). Cohrs, Kielmann, et al. [45] also show that a strong desire for personal freedom and autonomy negatively predict endorsement of restraints on civil liberties. Thus, we hypothesised that endorsement of liberty would be strongly negatively associated with support for potential surveillance technologies during the Covid-19 pandemic.

We also considered the role of political views (liberal or conservative), which have been studied as predictors of attitudes towards restrictions on rights and civil liberties [49, 52]. Political conservatism, which is closely associated with RWA, has already been shown to be related to a willingness to restrict civil liberties and to attitudes against freedom of movement [56–58].

Because gender is correlated with risk taking and protective behaviour [59–61] and age is related to attitudes towards new technologies [62, 63], in both studies, we examined our models with gender and age as control variables.

## 6. Overview of the studies

In this research, we examined the feelings related to the Covid-19 pandemic and ideological beliefs as predictors of support for the surveillance technologies that are currently being discussed as posing a potential threat to individual rights. To our knowledge, this is the first research that examines whether factors related to the pandemic situation (perceived personal threat and lack of control) are stronger predictors of surveillance technologies than individual factors related to personal values and ideological beliefs.

We conducted two studies among Polish citizens. In Study 1 we tested a hypothesis that lower sense of personal control would be associated with an increased acceptance of such technologies because they have the potential to restore order and normality and we also expected that a high personal threat would be positively related to support for such technologies because they might sufficiently reduce the risk of being infected.

In Study 2, we intended to replicate the results of Study 1 and furthermore we tested the role of ideological beliefs, RWA and individual endorsement of liberty, in predicting the acceptance of surveillance technologies, hypothesising that RWA would be positively and endorsement of liberty negatively related to the acceptance of such measures. We also tested whether feelings related to the pandemic would be still valid predictors of acceptance of surveillance technologies when RWA and endorsement of liberty were included in the model. In both studies, we also controlled for political views.

In these two studies, we used a different operationalization of the dependent variable. In Study 1, due to a lack of space, we needed to use a single item measure while, in Study 2, we developed a longer scale to measure attitudes towards the tracking technologies counteracting the pandemic.

The studies were approved by the Scientific Research Ethics Committee of the Faculty of Psychology, University of Warsaw.

## 7. Study 1

In the first study, using a correlational design, we tested the extent to which perceived level of personal control regarding the coronavirus pandemic was related to support for radical measures for counteracting the pandemic. The study was conducted in Poland, where the first case of Covid-19 was diagnosed on March 4. On March 12, the Polish government announced the closure of schools, shopping malls and restaurants and recommended social distancing. The study was done from March 13 through March 15, during the period in which the first restrictions were introduced.

### 7.1 Method

#### 7.1.1 Participants and procedure

Study 1 was a part of broader research conducted on a nationwide sample (N = 1,046) via an online research panel. The panel’s participants were volunteers who participate in surveys for small material rewards). We used quota sampling with quotas based on the gender, age and size of place of residence of the general Polish adult population (aged 18–70). Women accounted for 52% of the sample, and the mean age was 44.35 years (*SD* = 14.63). All the participants provided informed consent to take part in the research by clicking the button “I agree” after reading information about the study.

#### 7.1.2 Measures

*Support for radical measures to counteract the pandemic*. We asked the participants to indicate their agreement with a single face-valid item: ‘Authorities should have the right to control with sensors and applications the movement of citizens’ (1 = *strongly disagree* to 7 = *strongly agree*).

**Lack of personal control** was measured by three items referring to the coronavirus pandemic, e.g., ‘The coronavirus outbreak has made me feel less in control in my life’ (see also [64]). The participants were asked to answer on a 7-point scale (1 = *strongly disagree* to 7 = *strongly agree*) (α = .77). We used the mean value of three items in the analyses.

**Personal threat** was measured by two items related to the personal risk of being infected by coronavirus, e.g., ‘I’m afraid I could get infected and get sick’. Participants answered ‘yes’ or ‘no’ to these questions. We used the mean value of two items in the analyses.

We controlled for **moral conservatism**, which was measured by seven items on attitudes towards abortion, euthanasia, same-sex marriages, in-vitro procedures and contraception usage. The participants responded on a scale from 1 (*strongly disagree*) to 4 (*strongly agree*) (α = .90). The measure was based on the scale previously used in Maison, [65, 66].

The full scales are provided in the S1 Appendix.

### 7.2 Results & discussion

#### 7.2.1 Zero-order correlations

The correlations between the main variables of both studies are presented in Table 1. Both lack of control and moral conservatism were positively correlated with support for radical measures to counteract the pandemic.

**Table 1:** Means, standard deviations, and correlations between main variables (Study 1).

	M	SD	95% CI	2	3	4
1. Lack of personal control	4.36	1.31	[4.28, 4.44]	.30**	-.01	.26**
2. Support for radical measures counteracting pandemic	3.92	1.97	[3.80, 4.04]		.15**	.16**
3. Moral conservatism	2.16	0.78	[2.11, 2.20]			-.05
4. Personal threat	0.49	0.41	[0.46, 0.51]			

* *p* < .05

** *p* < .01

#### 7.2.2 Regression analysis

The results of the multiple regression analysis showed that both personal threat and lack of personal control predicted support for radical measures to counteract the pandemic The results did not change significantly when controlled for moral conservatism, gender and age. Moral conservatism and age were also significant predictors of support for tracking technologies (see Table 2).

**Table 2:** Regression analysis of attitudes towards radical measures to counteract the pandemic (N = 1033).

Dependent variable	Attitudes towards radical measures to counteract the pandemic			
Predictors	B (SE)	β	B (SE)	β
Constant	1.90 (.20)**		1.37 (.32)**	
Lack of control	0.42 (.05)	0.28**	0.42 (.05)	0.28**
Personal threat	0.41 (.15)	0.09**	0.47 (.15)	0.10**
Moral conservatism			0.40 (.07)	0.16**
Gender (1−Women, 0 − Men)			0.06 (.12)	0.02
Age			-0.01 (.04)	-0.06*
R^2^	0.10		0.13	

* *p* < .05

** *p* < .01

Study 1 confirmed our hypothesis that personal threat and lack of personal control are positively related to higher support for technologies that track citizens to fight the spread of coronavirus.

## 8. Study 2

In the second study, we examined whether the two predictors included in Study 1 as well as ideological views, such as RWA and endorsement of liberty, would significantly predict attitudes towards surveillance technologies to counteract the pandemic.

Study 2 was a part of broader research conducted in Poland from 19 through 24 March during a period in which further cases of Covid-19 were diagnosed. On March 20, the state of epidemic was introduced in Poland, giving the state authorities new entitlements. Free movement was gradually restricted, and private gatherings were banned. The government also introduced the possibility of using an application to monitor compliance with the home quarantine. The application, which uses geo-location and facial recognition algorithms, became mandatory on April 1.

### 8.1 Method

#### 8.1.1 Participants and procedure

We intended to recruit at least 1,000 people before March 24 because, the next day, the Polish government planned to introduce further restrictions. In the end, 1,680 persons (74% women) participated in an online study conducted on Facebook. The information about our research was distributed on several Facebook pages and groups. The full text of the Facebook post is included in the S1 Appendix. Participants were offered a possibility of remuneration (participation in a drawing of five vouchers worth c.a. $13). The sample consisted of people aged 18–69 (*M* = 26.20, *SD* = 6.95). All the measures were presented in randomised order and some participants withdrew before the end of questionnaire which is why the number of participants differs for various measures. All the participants provided informed consent to take part in the research by clicking the button “I agree” after reading information about the study.

#### 8.1.2 Measures

*Attitudes towards surveillance technologies to counteract the pandemic*. Because no existing measure could be found that assessed support for surveillance technologies related to the current pandemic, we designed items based on solutions that have been introduced in several countries.

The measure consisted of five items related to surveillance technologies, e.g., ‘Surveillance cameras with an automatic facial recognition system to quickly identify persons who do not comply with the authorities’ recommendations’ (α = .78). We asked the participants to indicate their attitude towards these technological solutions on a scale from 1 (*It definitely shouldn’t be introduced in Poland*) to 7 (*It definitely should be introduced in Poland*). We used the mean value of five items in the analyses.

**Lack of personal control** was measured using the same scale as in Study 1 (α = .68).

**Personal threat** was measured with three items related to the personal risk of being infected and becoming ill, e.g., ‘I consider the risk of personally getting Covid-19 to be high’ (α = .66). We used the mean value of three items in the analyses.

**Endorsement of liberty** was measured with one item: ‘The freedom to do what we want is more important than following the recommendations of the authorities’ designed for the purpose of this study. The participants responded on a scale from 1 (*strongly disagree*) to 7 (*strongly agree*).

**Right-wing authoritarianism** was measured with four items related to authoritarian aggression and submission based on Funke's scale [67] e.g., ‘What our country really needs is a strong, determined leader who will crush evil and take us back to our true path’ (see also [68]). We decided to include questions related to submission and authorities because conservatism was measured separately (as political views). Participants responded on a scale from 1 (*strongly disagree*) to 7 (*strongly agree*) (α = 0.66). We used the mean value of four items in the analyses.

**Political views** were measured with two items: ‘What are your moral views?’ and ‘What are your economic views?’ (see e.g., [69, 70].Participants responded on a scale from 1 (*conservative*) to 7 (*liberal*) for moral views and on a scale from 1 (*liberal*) to 7 (*social*) for economic views.

The full scales are provided in the S1 Appendix.

### 8.2 Results & discussion

#### 8.2.1 Measurement model

A confirmatory factor analysis in MPLUS (version 7, [71]) showed that attitudes towards surveillance technologies, RWA, perceived threat, and lack of control represent empirically distinct constructs (we excluded endorsement of liberty from this analysis since it was measured by one item). A model with four latent factors fitted the data well, χ^2^(84) = 317.57, *p* < .001, RMSEA = 0.04, CFI = 0.95, TLI = 0.93, SRMR = 0.04. The results of principal axis EFA also revealed four factors accounting for 17.96%, 13.23%, 12.85% and 11.99% of variability.

#### 8.2.2 Zero-order correlations

The correlations between the main variables of both studies are presented in Table 3. Perceived lack of control, personal threat and RWA were positively correlated and endorsement of liberty negatively correlated with support for surveillance technologies. Political views were not related to attitudes towards these technologies.

**Table 3:** Means, standard deviations, and correlations between main variables (Study 2).

	M	SD	95% CI	2	3	4	5	6	7
1. Attitudes towards surveillance technologies (*N* = 1680)	4.31	1.45	[4.19, 4.34]	.16**	.30**	-.23**	-.02	.04	.26**
2. Lack of personal control (*N* = 1547)	4.04	1.50	[3.94, 4.10]		.05	-.01	.06*	.07**	.42**
3. RWA (*N* = 1404)	3.37	1.23	[3.30, 3.43]			-.08**	-.48**	-.05	.12**
4. Endorsement of liberty (*N* = 1526)	1.87	1.31	[1.78, 1.91]				-.02	-.12**	-.13**
5. Political views (moral) (*N* = 1680)	5.21	1.79	[5.13, 5.32]					.12**	-.01
6. Political views (economic) (*N* = 1680)	3.69	1.43	[3.62, 3.77]						.04
7. Personal threat (*N* = 1680)	3.35	1.42	[3.28, 3.42]						

* *p* < .05

** *p* < .01

#### 8.2.3 Regression analysis

The results of the multiple regression analysis demonstrated that both personal threat and lack of control were significant predictors of attitudes towards surveillance technologies, but personal threat seemed to be a stronger predictor. When RWA and endorsement of liberty were added, RWA appeared to be the strongest predictor of acceptance of such technologies (change in R^2^ was significant at p < .001). The main pattern of results remained the same when we added political views as control variables. Gender was significantly related to attitudes towards surveillance technologies with women being more in favor of them than men. See Table 4 for the exact coefficients.

**Table 4:** Regression analysis of attitudes towards surveillance technologies (N = 1404).

Dependent variable	Attitudes towards surveillance technologies to counteract the pandemic					
Predictors	B (SE)	β	B (SE)	β	B (SE)	β
Constant	3.29 (.12)**		2.83 (.15)**		1.80 (.27)**	
Lack of control	0.06 (.03)	0.06*	0.07 (.03)	0.07**	0.05 (.03)	0.05^†^
Personal threat	0.23 (.03)	0.22**	0.16 (.03)	0.16**	0.15 (.03)	0.15**
RWA			0.30 (.03)	0.26**	0.38 (.03)	0.33**
Endorsement of liberty			-0.22 (.03)	0.19**	-0.20 (.03)	-0.17**
Political views (moral)					0.11 (.02)	0.13**
Political views (economic)					-0.03 (.03)	-0.03
Gender (1−Women, 0 − Men)					0.29 (.08)	0.09*
Age					0.01 (.01)	0.03
R^2^	0.06		0.17		0.20	

^†^ *p* = .05

* *p* < .05

** *p* < .01

**Fig 1:**
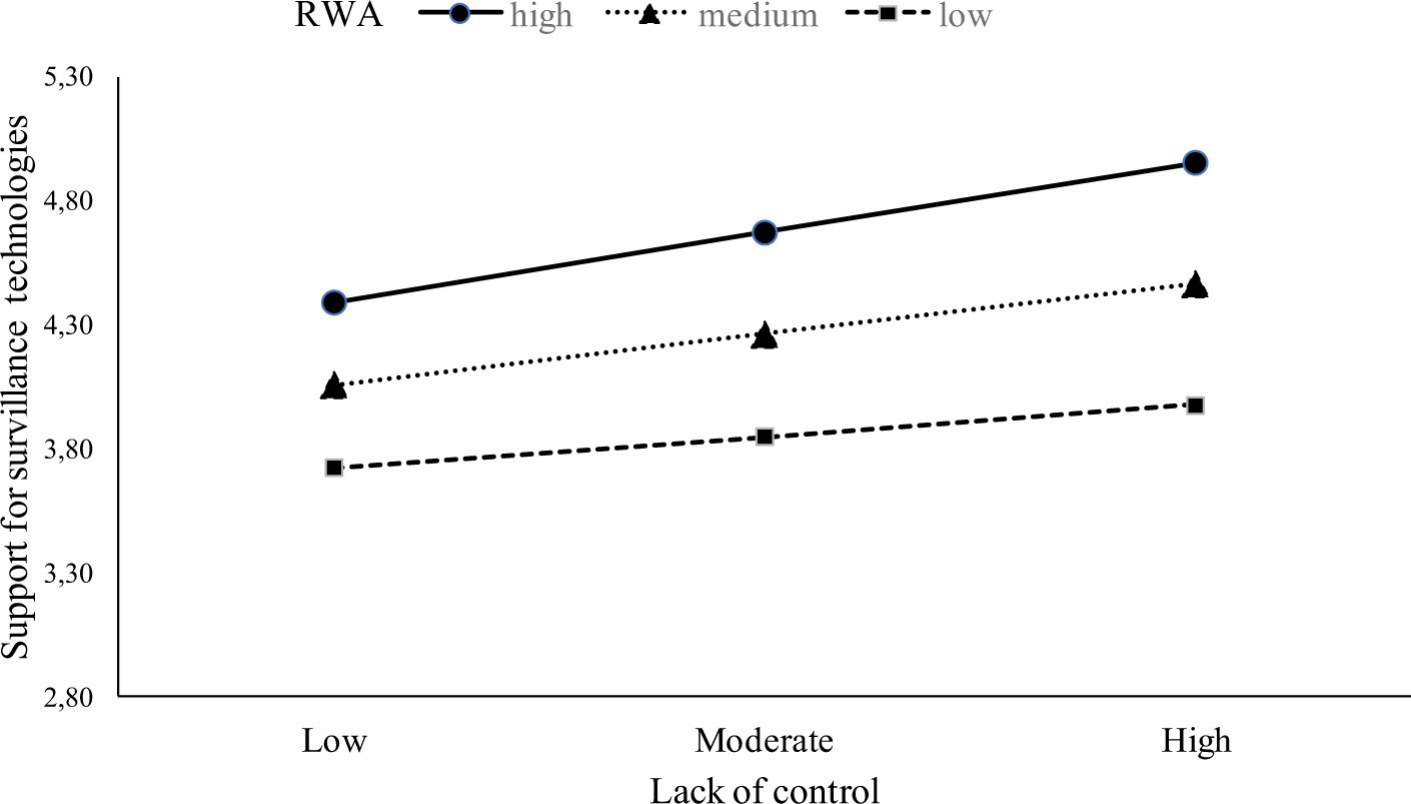
Moderating effect of RWA and lack of control on support for surveillance technologies.

To test our hypotheses about moderating role of RWA we used macro PROCESS, model 1. In line with predictions, the relation between lack of control and attitudes towards surveillance was strengthen by RWA, *B =* 0.04, *SE =* 0.02, p = 0.02 (assuming RWA at + 1 *SD*: *B* = 0.19; *SE* = 0.03; *p <* .001; assuming RWA at −1 *SD*: *B* = 0.08; *SE* = 0.03; *p =* .01; see Fig 1). We further used the Johnson-Neyman technique to probe for interaction and to identify ranges of values of the moderator for which the interaction effect is significant. It showed that lack of control significantly predicted acceptance of surveillance technology for level of RWA higher than 1.84.

**Fig 2:**
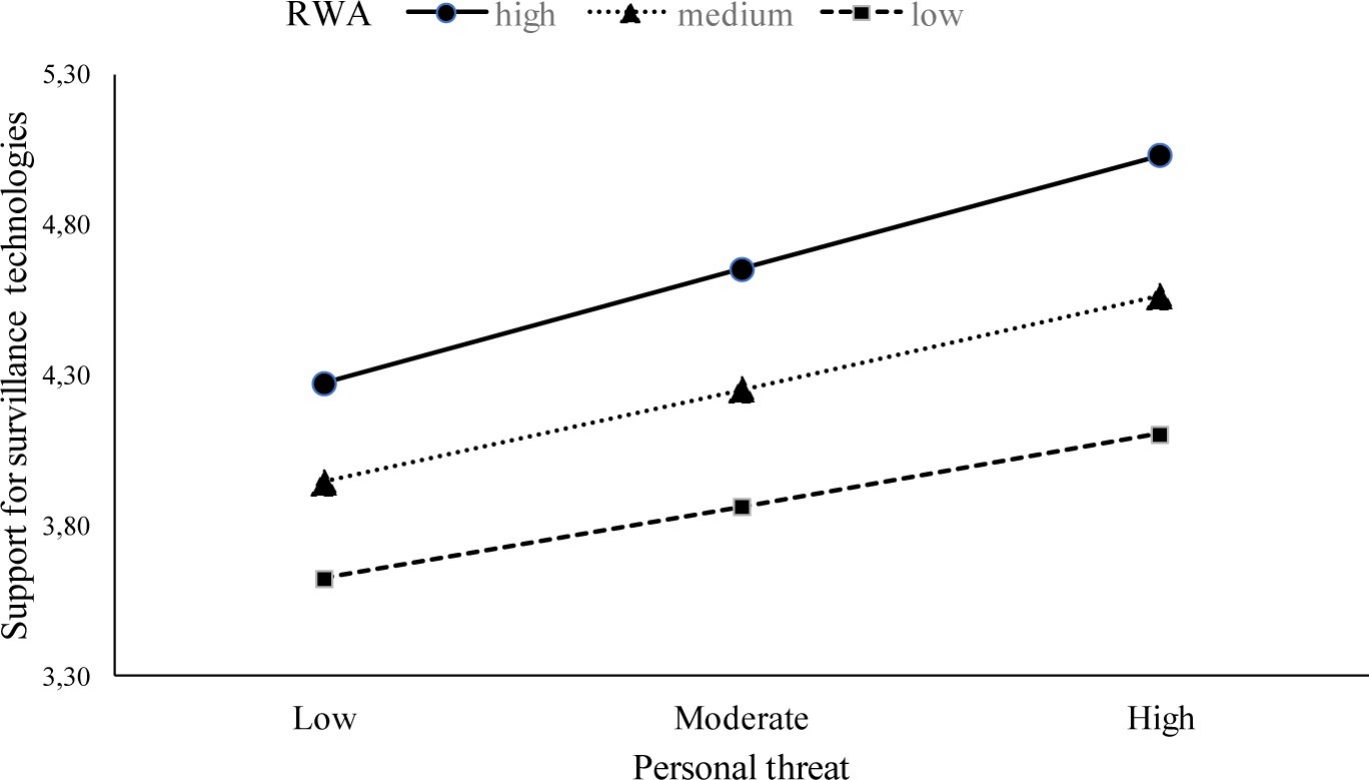
Moderating effect of RWA and personal threat on support for surveillance technologies.

Similarly, RWA and personal threat interacted negatively, *B =* 0.04, *SE =* 0.02, p = 0.04. Personal threat was positively related to support for surveillance technologies among those participants who declared high level of RWA (assuming RWA at + 1 *SD*: *B* = 0.27; *SE* = 0.04; *p <* .001). The relation between perceived threat and support for surveillance technologies for people low on RWA was weaker (assuming RWA at −1 *SD*: *B* = 0.17; *SE* = 0.04; *p <* .001; see Fig 2).

The results of Study 2 confirm our main hypotheses that both feelings related to the pandemic and ideological views are significantly correlated with support for surveillance technologies; however, ideological views appear to be stronger predictors than variables related to the pandemic.

## 9. General discussion

The sudden outbreak of the coronavirus pandemic has led to high levels of uncertainty in society. Decisions on how to proceed in handling the situation are made on an ongoing basis, often without access to sufficient data. Almost every week, important decisions are made that affect the everyday lives of citizens who suddenly find themselves in a new reality with remote work and schooling and restrictions on movement. This causes feelings of uncertainty and lack of control and thus a natural desire to overcome that state (see e.g., [3]). Additionally, perceptions of one’s own lack of control can be accompanied by justified concerns about one’s life and health.

Our research examined the extent to which situational perception of lack of control and the individual threat of being infected and ideological beliefs (authoritarianism and endorsement of liberty) were associated with greater acceptance of technological solutions that both aim to mitigate the coronavirus pandemic and simultaneously pose a potential threat to privacy and other civil rights.

We argued that, in line with the compensatory control model [17], such technologies by their very nature can help to restore the impression that there are strict standards and rules and a need to comply with them. Thus, these restrictive measures can help in dealing with the uncertain and dynamically changing situation of a pandemic, particularly if someone perceives a lack of personal control. Moreover, based on previous research on terrorist threats [45, 50], we expected that a higher personal threat would be related to greater support for technological surveillance. Our results show that, although both lack of control and perceived threat significantly predict the acceptance of surveillance technologies, perceived threat had a greater explanatory value, while ideological beliefs were included. It seems, therefore, that a perceived realistic threat to life or health during a pandemic is a more important predictor of acceptance of potentially helpful yet controversial technologies than a sense of uncertainty and unpredictability in times of crisis. These results extend the findings of Kay et al. [17] to two new contexts: (1) modern technology as a potential new method for indirect restoration of control but posing a threat to privacy and (2) the extraordinary global crisis.

The second main objective of our study was to investigate how the acceptance of surveillance technologies is related to ideological beliefs: RWA and the endorsement of liberty. RWA and the endorsement of liberty were both associated (whether positively or negatively) with acceptance of these technologies, and both were stronger predictors than the characteristics related to the Covid-19 pandemic. In line with previous studies, which have shown that those who appreciate freedom are particularly guided by that value in assessing moral issues [55], high endorsement of liberty predicted less positive attitudes towards the implementation of surveillance technologies. In turn, RWA was a positive predictor of acceptance of these technologies as in previous studies on the possible trade-off between civil rights and security under terrorist threats [51]. Moreover, as previous research has shown that RWA is particularly related to the need for safety and risk minimisation [43, 44], we examined whether RWA moderated the relationship between both perceived threat and lack of control and acceptance of surveillance technologies. These two relationships were indeed particularly apparent among people high in authoritarianism, who, when feeling a lack of control or a coronavirus-related threat, have more positive attitudes towards such technologies.

In Study 2, we also found that women are more likely than men to accept surveillance technologies. This is in line with evidence in the literature showing that women are usually risk averse and take more precautions than men [59, 60]. A recent study has shown, for example, that women more than men intended to wear a face mask during the Covid-19 pandemic and that men believe it is shameful to wear a face covering [61]. However, while this effect was seen in Study 2, it did not appear in Study 1, which featured a more balanced group of participants in terms of gender and age. It seems, though, that young men (who were the majority of male participants in Study 2) may feel that needing any measure to protect them from coronavirus is a sign of weakness (see [61] for a similar effect).

To sum up, our studies show that variables related to the situational context (such as situational lack of control and perceived threat to individual health and life) significantly predict the acceptance of technologies used to combat the pandemic; nevertheless, the strongest predictors were variables related to more stable individual features, i.e., RWA and the endorsement of liberty. To our knowledge, this study represents the first research on the predictors of acceptance of surveillance technology used to mitigate the Covid-19 pandemic and fills the gap in the existing research on crises, showing that individual ideological beliefs are stronger predictors of attitudes towards surveillance than variables related to threat to life and uncontrollability of the situation. In contrast to previous studies that focused mostly on terroristic threats, our study focused on the danger of a much larger scale and less controllable event, namely, a global pandemic. The research was ecologically valid as we asked about technologies that have already been or have begun to be implemented in other countries. Moreover, by conducting the research at two time points (shortly after the first major restriction and 10 days later), we showed that the expected relationships between our variables are stable (however, it is important to note that this was at a relatively early stage of the pandemic in Poland).

Future studies are needed to further develop the results. First, it would be important to determine how far citizens are willing to accept such technological solutions even after the pandemic and, in particular, whether getting used to their presence during a ‘state of exception’ has a mitigating effect on possible concerns in the future.

Additionally, our results can provide the basis for important experimental studies. In particular, it is worth investigating whether the experimental manipulation of the sense of control has an impact on increased acceptance of new technologies that potentially threaten privacy. Another interesting study would involve verifying the extent to which acceptance of these surveillance technologies depends on presenting them as temporary or permanent.

### 9.1 Limitations and future directions

We should stress some limitations of this research. First, both our studies were correlational, so they do not establish causal relationships between the variables; however, it seems more plausible that lack of control, personal threat, RWA and endorsement of liberty predicted support for radical measures counteracting the pandemic than the reverse (see e.g., Carriere, 2019). Second, we did not measure the perceived efficacy of these surveillance technologies, and it would be worth examining directly whether the introduction of such solutions would, in fact, restore the sense of personal control and reduce the sense of threat.

Our study differed in terms of sample characteristics, Study 1 was based on a representative sample of Polish and in Study 2, women and young people were overrepresented, so replication in a more gender- and age-balanced sample is required, however, we have shown that the main relationships between IVs and DVs hold true over and above the effects of gender and age. Another limitation of our studies is that some variables were measured by relatively short scales.

It is possible that these results are more representative for countries that have not yet introduced such technological measures to fight the coronavirus pandemic. Future research could include citizens from countries that have already used such technologies and could also compare states with stricter (e.g., China, Taiwan) and more relaxed (e.g., Sweden) policies regarding the coronavirus pandemic.

### 9.2 Conclusion

Epidemiologists warn against the next wave of the Covid-19 pandemic and future similar epidemic crisis [72, 73]. We may expect that many countries will be willing to introduce tracking technologies to counteract the epidemics, hence, understanding people's attitudes towards these technologies seems extremely important. Our studies suggest that negative feelings evoked by the Covid-19 pandemic may not be as important predictors of these attitudes as people's general viewpoints on submission to authority and civil freedoms.

## Supporting information

S1 Appendix (DOCX)

S1 Data (RAR)
