# Supplementary material for: The acceptance of Covid-19 tracking technologies: The role of perceived threat, lack of control, and ideological beliefs
Source: PLoS One. 2020 Sep 11;15(9):e0238973. doi: 10.1371/journal.pone.0238973 (PMC7485859; doi:10.1371/journal.pone.0238973)
Supplement: S1 Appendix — (DOCX) [file pone.0238973.s001.docx]

**Appendix**

Measurement tools used in Studies 1 & 2

**Lack of personal control (Studies 1 & 2)**

The coronavirus outbreak has made me feel less in control in my life

I feel like whatever I do, I won't be able to protect myself effectively against the coronavirus.

The coronavirus pandemic has made me uncertain what will happen to me in the near future.

**Personal threat**

I’m afraid I could get infected and get sick (Study 1).

I’m afraid that one of my loved ones could get infected and get sick (Study 1).

I consider the risk of personally getting Covid-19 to be high (Study 2).

I’m afraid that, if I were infected, the disease would be severe (Study 2).

I think there’s a great risk that I’ll get the coronavirus from people whom I am in contact with (Study 2).

**Right-wing authoritarianism (Study 2)**

What our country really needs is a strong, determined leader who will crush evil and take us back to our true path.

The real keys to the ‘good life’ are obedience and discipline.

The withdrawal from tradition will turn out to be a fatal fault one day.

Obedience and respect for authority are the most important values children should learn.

**Endorsement of liberty (Study 2)**

The freedom to do what we want is more important than following the recommendations of the authorities

**Moral conservatism (Study 1)**

Abortion (termination of pregnancy) should be allowed.
Euthanasia (taking life on request) should be allowed.
People should have the right to be in same-sex relationships.
Homosexual couples should have the right to marry.
The state should support couples in accessing in-vitro fertilization.
People should be allowed to use contraceptives if they want to.
People should have access to and the right to use abortion pills.

**Political views (Study 2)**

What are your moral views?

What are your economic views?

**Support for radical measures to counteract the pandemic (Study 1)**

Authorities should have the right to control with sensors and applications the movement of citizens

**Attitudes towards surveillance technologies to counteract the pandemic (Study 2)**

Applications for government services to analyze private messages, to identify people potentially infected with the coronavirus.

Surveillance cameras with an automatic face recognition system to quickly identify persons who do not comply with the authorities' recommendations.

Surveillance cameras with body temperature detection system to determine where potentially sick people are.

Applications based on automatic location of users, informing them that they have been in places where there is a risk of contracting a coronavirus.

Applications that monitor compliance with home quarantine based on location

Facebook advertisement used in Study 2

Ladies and gentlemen,

We are psychologists from the University of Warsaw. We would like to invite you to take part in a study on feelings and opinions related to the coronavirus pandemic, Covid-19. The results will contribute to a better understanding of what we feel and how we react in this difficult period, so we appreciate your participation a lot!

The survey takes about 15–20 minutes. Participation is voluntary and we guarantee the confidentiality of all your answers. You may withdraw from the study at any time. As a token of appreciation for your time, participants who fill out the whole questionnaire take part in a lottery to win one of five EMPIK [the name of a Polish bookstore] vouchers worth 50 PLN [about $13].

In order to participate, please open the following link: xxx
